# An ancient Chinese wisdom for metabolic engineering: Yin-Yang

**DOI:** 10.1186/s12934-015-0219-3

**Published:** 2015-03-20

**Authors:** Stephen G Wu, Lian He, Qingzhao Wang, Yinjie J Tang

**Affiliations:** Department of Energy, Environmental and Chemical Engineering, Washington University, St. Louis, MO 63130 USA; Fine Chemicals & Biocatalysis Research, BASF Corporation, Tarrytown, NY 10591 USA

**Keywords:** ATP, Energy metabolism, Flux analysis, Free energy, Maintenance loss, Semi-biosynthesis

## Abstract

In ancient Chinese philosophy, Yin-Yang describes two contrary forces that are interconnected and interdependent. This concept also holds true in microbial cell factories, where Yin represents energy metabolism in the form of ATP, and Yang represents carbon metabolism. Current biotechnology can effectively edit the microbial genome or introduce novel enzymes to redirect carbon fluxes. On the other hand, microbial metabolism loses significant free energy as heat when converting sugar into ATP; while maintenance energy expenditures further aggravate ATP shortage. The limitation of cell “powerhouse” prevents hosts from achieving high carbon yields and rates. Via an *Escherichia coli* flux balance analysis model, we further demonstrate the penalty of ATP cost on biofuel synthesis. To ensure cell powerhouse being sufficient in microbial cell factories, we propose five principles: 1. Take advantage of native pathways for product synthesis. 2. Pursue biosynthesis relying only on pathways or genetic parts without significant ATP burden. 3. Combine microbial production with chemical conversions (semi-biosynthesis) to reduce biosynthesis steps. 4. Create “minimal cells” or use non-model microbial hosts with higher energy fitness. 5. Develop a photosynthesis chassis that can utilize light energy and cheap carbon feedstocks. Meanwhile, metabolic flux analysis can be used to quantify both carbon and energy metabolisms. The fluxomics results are essential to evaluate the industrial potential of laboratory strains, avoiding false starts and dead ends during metabolic engineering.

## Introduction

In the past decade, molecular biology tools have been developed rapidly and now offer new opportunities for metabolic engineering of microbial hosts [1-6]. These tools include the selection of plasmids with different copy numbers, promoter engineering, codon optimization, synthetic scaffolds, directed evolution or rational design of enzymes, ribosome binding sites editing, and competitive pathways deletion. Advanced genome engineering (e.g., CRISPRs and TALENs) and automation of conventional genetic techniques (e.g., MAGE) provide efficient capabilities for editing genomes and evolving new functions. At the same time, systems biology (e.g., genomics, transcriptomics, and proteomics) can characterize complex cell networks, mine useful genes, discover new enzymes, reveal metabolic regulations, and screen mutant phenotypes. The advent of these powerful tools seems to lead researchers into a new epoch of bioprocess industries using GMMs (genetically modified microorganisms) in the near future. However, that is not the whole story.

The golden age of industrial biotechnology dawned in the early 1940s, driven by the mass production of penicillin and enjoyed a fast growth in the 1950s ~ 1980s. Microbial bioprocess has produced diverse commodity chemicals (such as ethanol, amino acids, citric acid, and lactate) as well as recombinant proteins and antibiotics in the last century. Those commercial products mainly rely on natural strains or strains with minor genetic modifications (usually only one or few new genes). Since the recent decade, in the hope of producing chemicals at low costs and reducing greenhouse gas emissions, an enormous amount of investment has been devoted to metabolic engineering in many nations. Although modern biotechnologies can engineer microbial platforms to synthesize diverse products in laboratories, there are only a few GMM products that have become commercially promising in the past decade (e.g., artemisinic acid and 1, 4-butanediol). Novel GMMs are also used for chemical manufactures, such as short-chain alcohols and isoprene [7-9]. Recently, Gevo and Butamax introduce the keto-acid/Ehrlich pathway into yeasts to produce isobutanol [10]. Amyris extend the mevalonate pathway in *Saccharomyces cerevisiae* for branched and cyclic terpenes (e.g., farnesene) synthesis. However, these companies have not achieved strong net profit margin yet. To date, the industrial-scale biofuel is still ethanol, which is cheaply manufactured from sugar cane in Brazil. In this perspective, we address one of the hidden constraints in microbial cell factories (i.e., energy metabolism).

## The energy losses in microbial cell factories

**Figure 1:**
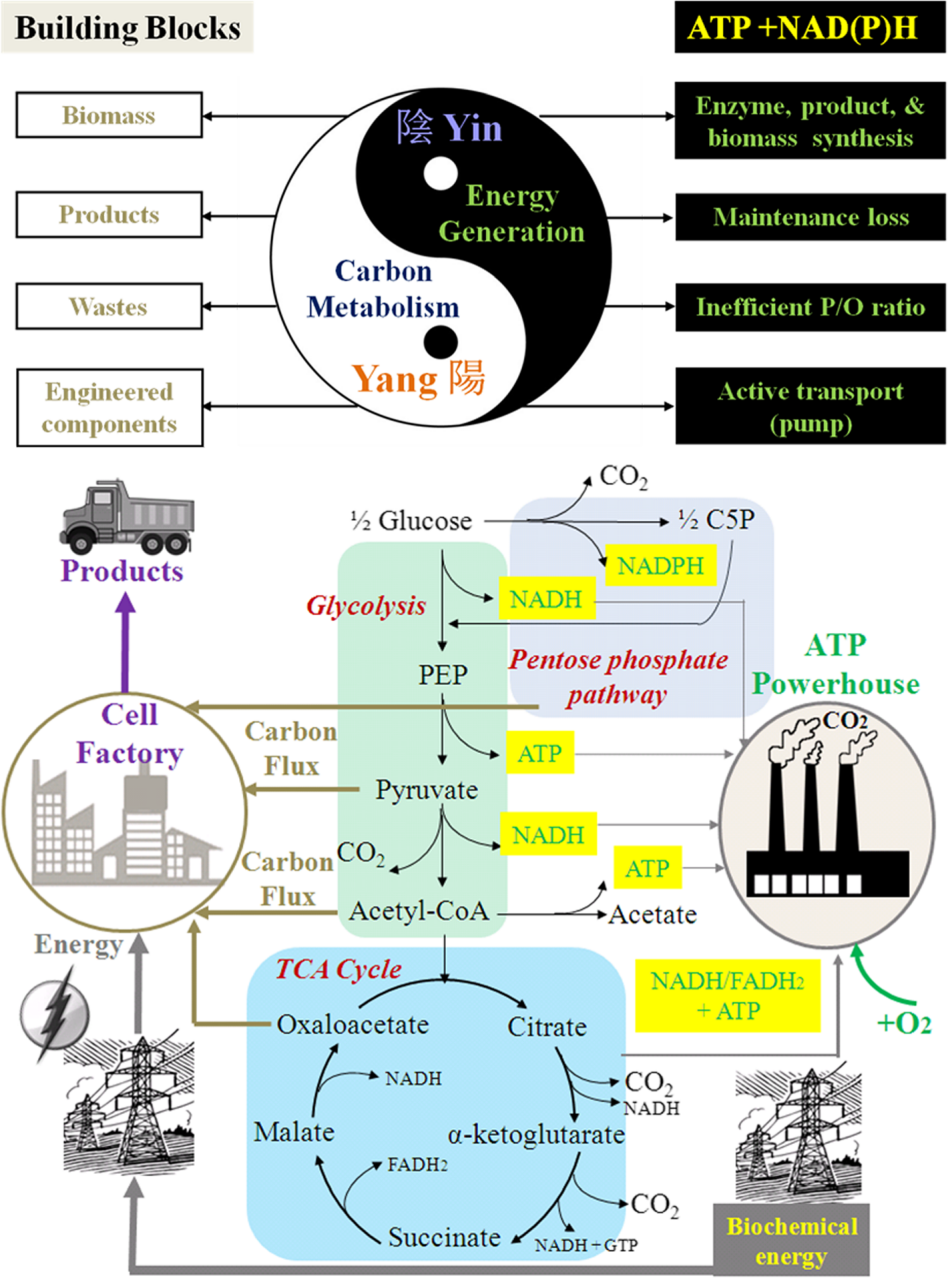
**Cell carbon and energy metabolism illustrated by Yin-Yang Theory (note: engineered components include plasmids, over-expressed enzymes, synthetic circuits, etc.).**

Heterotrophic organisms obtain free energy in the form of ATP by breaking organic substrates into CO_2_ (Figure 1). Theoretically, oxidation of one mole of glucose to CO_2_ (∆_c_H^Θ^_298_ ≈ −2.8 MJ/mol) can generate 38 moles of ATP. Hydrolysis of these ATP to ADP (ΔG^Θ^ = −30.5 kJ/mol) provide ~1.2 MJ of biochemical energy. Thereby, ~60% of energy from glucose is lost as heat during ATP synthesis (similar to a Carnot heat engine). Besides, cell consumes ATP for diverse maintenance activities, such as nutrient/metabolite transport, chemotaxis, chemical gradient preservation, biomass component repair, and macromolecule re-synthesis [11]. These maintenance costs, essential for cell survival and stress adaptation, compete for ATP resources for biomass growth and product synthesis.

Microbial hosts have not evolved towards optimal energy metabolism. Over billions of years of evolution, microbes with a higher growth rate gained a selective advantage when competing for shared energy resources, but these fast growing species have a lower yield of ATP from substrates (e.g., less than 30 ATP/glucose) [12]. The oxidative phosphorylation (P/O) ratio represents ATP generation efficiency through substrate oxidation. Theoretically, three ATP can be obtained from the reduction of one oxygen atom (i.e., P/O = 3) during oxidative phosphorylation. Although slow-growing mammalian cells can achieve P/O values close to 3, bacteria and yeasts often have P/O ratios below 2.5 (note: microbes may dissipate the proton gradient before it can be fully used for charging the ATP synthase). In addition, microbial hosts may lose ATP yield due to byproducts synthesis, membrane leakage, removal of reactive oxygen species, or suboptimal cultivations (insufficient mixing, shear stress, or biofilm formation). Lastly, the electron transport chain for ATP generation and nutrient transporters may compete for membrane and intracellular spaces so that the capacity of the microbial powerhouse cannot be easily upgraded [13,14].

We introduce a terminology “metabolic entropy” to define the free energy in the substrates that is lost through energy metabolism and becomes unavailable for biosynthesis. Metabolic entropy has gained attention from metabolic flux analysis researchers because the objective function of biomass production in FBA (flux balance analysis) always overestimates microbial growth rates. Moreover, FBA predictions highly depend on the assumption of a fixed ATP maintenance coefficient. To address this problem, researchers developed ^13^C-metabolic flux analysis (MFA) to quantify the microbial “metabolic entropy” directly via tracer experiments. By examining *Bacillus subtilis* mutants, ^13^C-MFA has discovered that the suboptimal cell metabolism is associated with the increased energy usage in the face of environmental and random genetic perturbations [15]. This study suggests that mutating regulatory genes can drive carbon flow towards the desired pathways; however, hijacking carbon fluxes may sacrifice cell energy fitness for adaptive responses under adverse environmental conditions.

## The tradeoff between product yield and energy fitness

Traditional metabolic engineering uses plasmids and heterologous enzymes to redirect carbon fluxes. Early studies have shown high copy number plasmids cause significant alterations in cell properties and strongly influence metabolic engineering endeavors [16]. ^13^C-MFA of *E. coli* strains revealed higher acetate production and O_2_ uptake rates in plasmid-containing strains than in the plasmid-free strains [17]. The presence of plasmids can increase cell maintenance, decrease growth rate and change intracellular fluxes, especially suppressing the oxidative pentose phosphate pathway [18]. Similarly, synthetic biology parts (such as novel pathways, protein scaffolds, and genetic circuits) may also increase metabolic entropy if extra nucleic acids and proteins are required to be made by the hosts (note: elongation of one amino acid costs four ATP molecules) [19]. Natural microbes have frugal enzymatic machinery (each native enzyme in a single *E. coli* cell may only have dozens of molecule copies and places minimal biosynthesis burden on cell metabolism) [20]. During pathway engineering, a large portion of over-expressed enzymes may be inactivated due to protein misfolding. Considerable ATP expenditure for heterologous enzyme over-synthesis can trigger stress responses. For example, ^13^C-MFA has been used to examine metabolic burdens in *E. coli* during biosynthesis of recombinant proteins. The results indicate a 25% increase in the total ATP expenditure rate in the highest yielding strain (up to 45 mmol ATP/g CDW/h) [21]. To overcome such an energy limitation, *E. coli* has to reduce biomass synthesis and enhance oxidative phosphorylation for ATP generation. Besides, engineered microbial hosts often suffer from increased non-growth associated maintenance as well as reduced respiration efficiency (poor P/O ratio) due to membrane stresses [22,23]. If an extended heterologous pathway causes deleterious effects on carbon and energy metabolism, the host will lose the capability to grow in a minimal carbohydrate medium. In this case, rich nutrients, such as yeast extract (producing 1 g of yeast extract consumes >2 g of glucose), have to be supplied to relieve the cell’s metabolic burden [24].

Our theory of energy burden can guide strain development to tolerate product stresses. For instance, an isobutanol-tolerant mutant has been isolated after serial transfers; while the final isobutanol productivity of this evolved strain did not show improvement [25]. The export systems (e.g., ABC transporters) have been engineered for recovering cell growth under biofuel stresses [26], while ATP-driven efflux pumps show limited enhancement of short-chain alcohol productivity (~10%) [27]. Interestingly, efflux pumps are very effective when they are introduced into low-performance strains, in which their product titers are well below 1 g/L [26]. These observations explain the fact that cell stress adaptation requires ATP expenditure and induce energy burdens [28]. For the same reason, tolerance engineering often works well on yeast strains for ethanol production because of simple ethanol synthesis pathway and net ATP generation from glycolysis. For example, engineering transcriptional machinery or up-regulation of the potassium/proton pumps in *Saccharomyces cerevisiae* can improve both ethanol tolerance and the production titer (>100 g/L) [29,30]. In conclusion, if microbial hosts already have high metabolic burdens, tolerance engineering should focus on regulatory components rather than efflux pumps. For example, a methionine biosynthesis regulator can significantly improve both biofuel tolerance and productivity in *Escherichia coli* [27]. In yet another case, the inactivation of a histidine kinase may enhance the butanol productivity in *Clostridium acetobutylicum* by delaying cell sporulation [31].

## Sensitivity analysis of energy penalty on biofuel synthesis

**Figure 2:**
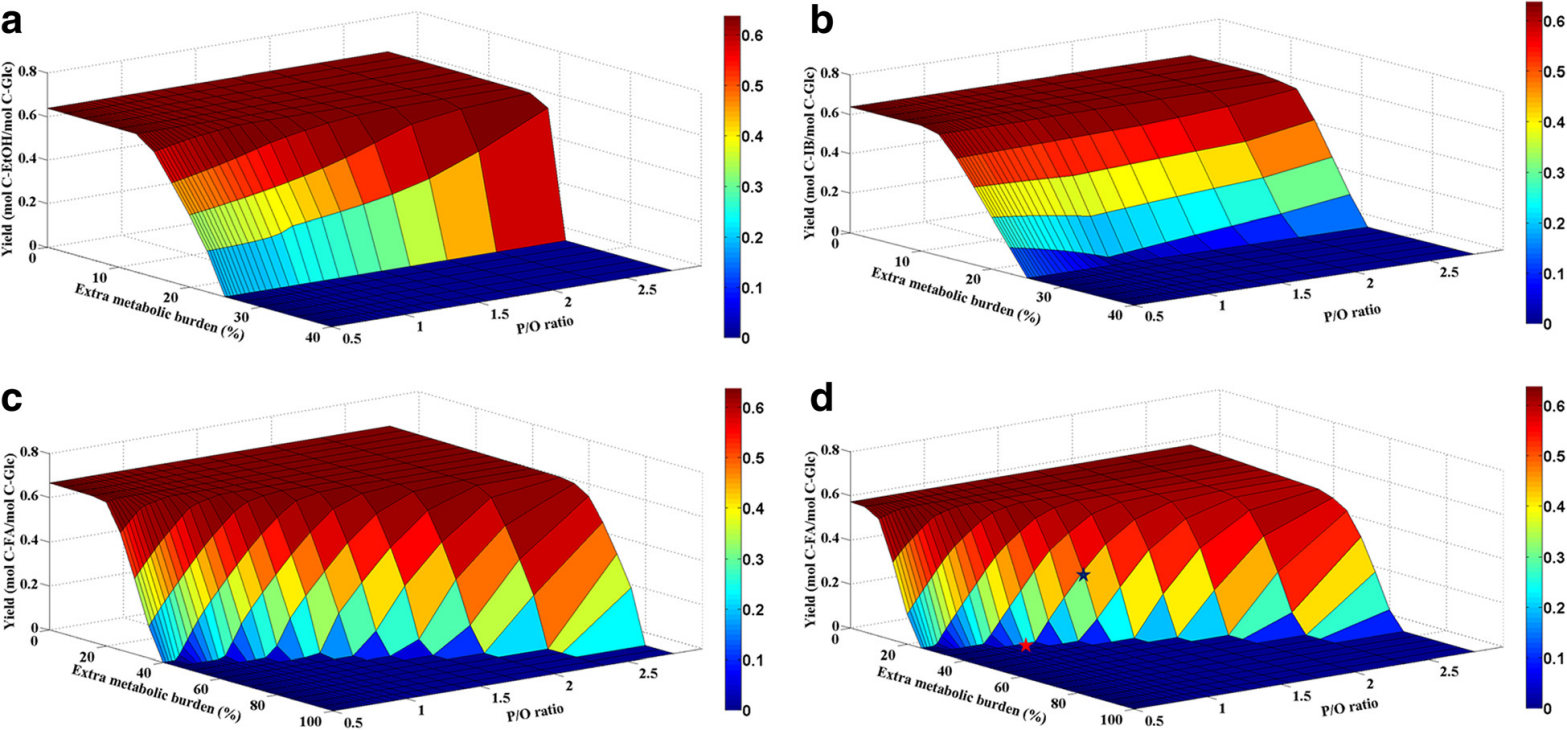
**Genome-scale FBA models for predicting microbial biofuel yields from glucose. a**. *E. coli* strains produce ethanol (growth rate = 0.05 h^−1^). **b.** *E. coli* strains produce isobutanol (growth rate = 0.05 h^−1^). **c**. *E. coli* strains produce fatty acid (growth rate = 0.05 h^−1^). **d**. *E. coli* strains produce fatty acid (growth rate = 0.20 h^−1^). We use an *E. coli* FBA model (iJO1366) to predict production of different biofuels from glucose. Alcohol production is simulated under the microaerobic condition (O_2_ influx ≤ 1.85 mmol/(gDW∙hr)), while fatty acid is under aerobic condition (O_2_ influx ≤ 12 mmol/ (gDW∙hr)). The medium conditions and glucose uptake rate (8 mmol/ (gDW∙hr)) are same for all FBAs. Extra metabolic burden is simulated by the costs of both protein overexpression and maintenance energy increase (e.g., 10% extra metabolic burden is equivalent to 10% overexpression of total biomass protein plus proportional increase of non-growth associated ATP loss). For each case, the objective function is set as to maximize the biofuel production. Abbreviations: DW (Dry Weight); FA (Fatty acid); Glc (Glucose); IB (Isobutanol).

We employ a genome-scale flux balance model (iJO1366) to simulate the adverse impacts of *E. coli* energy metabolism on biofuel product yields (Figure 2) [32]. Apart from the intracellular stress caused by enzyme overexpression, the release of large amounts of biofuel molecules (alcohol or fatty acid) will interfere enzymatic reactions *in vivo* and disrupt the cellular membrane’s integrity, which results in reduced efficiencies of oxidative respiration [25,33]. Thereby, metabolic engineering approaches are effective in redirecting carbon fluxes to biosynthesis only in these low-productivity strains whose energy metabolism are not overloaded. We use FBA to test the penalty of metabolic burdens (such as maintenance cost) and the decrease of P/O ratio on biofuel yields. The simulations show that microbial energy metabolism is usually abundant so that they can support certain amount of metabolic burdens without having apparent biosynthesis deficiency (e.g., without showing a slower growth after mutations). However, cell burden may increase during the routine genetic modifications. When cell powerhouse is unable to afford the increasing ATP expenditure, the biosynthesis yield will have a sudden drop (i.e., “the straw that broke the camel’s back”), forming a “cliff” in Figure 2.

FBA simulations yield two insights into microbial biofuels. First, alcohol (ethanol and isobutanol) producing *E. coli* strains not only have higher carbon yields (0.67 C-product/C-glucose), but also are insensitive to P/O ratios (Figure 2a, b). Comparing to isobutanol, ethanol production is less sensitive to the metabolic burden (larger energy sufficient zone). Ethanol fermentation, an ancient bioprocess from the beverage industry, does not need additional energy from O_2_, lowering its process costs. From a stoichiometric perspective, glycolysis generates two net ATP per glucose, which fulfills the cell energy expenditure. In addition, ethanol synthesis only needs a few native enzymes, and the hosts (e.g., *Saccharomyces cerevisiae*) are naturally tolerant to alcohols. The entire ethanol synthesis pathway is always inside of the cytosol, and thus they do not have mitochondrial transport limitations. These advantages explain why ethanol fermentation is superior to any other biofuel processes.

Second, energy metabolism may become a critical issue for synthesizing fatty acid-based compounds, which are susceptible to changes in P/O ratio, ATP maintenance loss, and oxygen uptake fluxes. Compared to alcohol production, fatty acid based fuels (such as biodiesel) require longer biosynthetic pathways (more enzymes to overexpress) and considerable ATP usage for product synthesis [34]. Besides, many enzymes in fatty acid pathway are tightly regulated during cell growth, leading to growth associated bio-production. The simultaneous biomass growth and fatty acid synthesis further exaggerates ATP shortage [35]. Therefore, aerobic fermentation has to be performed to enhance energy metabolism, which reduces product yield and increases the fermentation costs for aeration. Furthermore, the accumulation of fatty acid damages cell membrane and reduces oxidative phosphorylation efficiency. To demonstrate these synergistic effects on fatty acid yields, Figure 2c and d simulate *E. coli* fatty acid yields responding to P/O ratios and metabolic burdens. As shown in Figure 2c, fatty acid production can achieve a similar yield as ethanol if the host’s biomass growth rate (as 0.05 hr^−1^) and energy maintenance is not high. In reality, fatty acid yield can drop to 50% or less of the theoretical maximum, which is in consistent with the model prediction if we considered a practical biomass growth, extra ATP maintenance, and a low P/O ratio (<1.5) in FBA (blue star in Figure 2d) [36]. Figure 2d also indicates the high sensitivity of fatty acid yield in response to the P/O ratio (red star in Figure 2d). For instance, one unit change in P/O ratio leads to an abrupt drop in fatty acid yield -- from a theoretical maximum to zero.

## Yin-Yang theory in metabolic engineering

**Figure 3:**
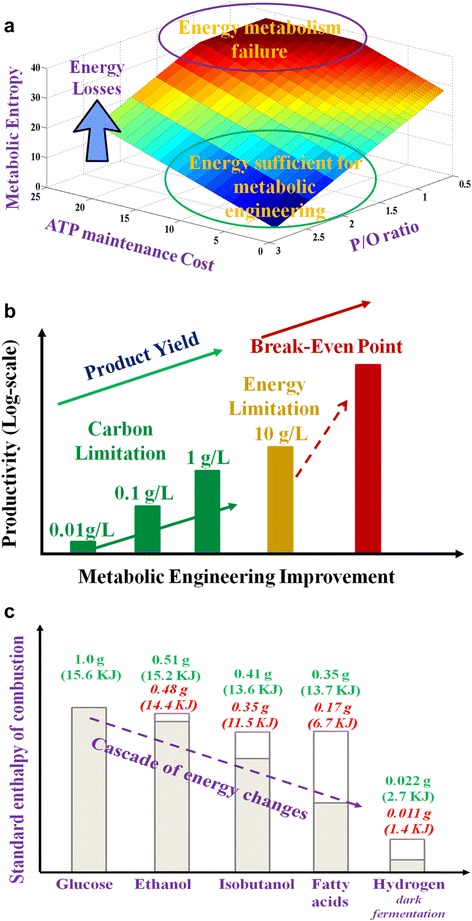
**Energy fitness and productivities in microbial cell factories. a**: The trend of metabolic entropy changes (unit: ATP generation per glucose). In optimal metabolism, one mole glucose generates 38 ATP for biosynthesis. Under constraints of P/O ratios and maintenance loss, less ATP can be generated (increase of metabolic entropy). **b**: The transition from carbon limitation to energy limitation with the increase of product yield. In many cases, the energy limitation prevents strains from achieving the yield and titer above break-even point. **c**: Cascade of energy changes (Heat of combustion) during biofuel synthesis from glucose. Energy was calculated based on stoichiometry yields (green text, entire bar) and the practical yields (red text, grey bar). Reported yields: ethanol -- 96% of theoretical yield [29], isobutanol -- 85% of theoretical yield [37], fatty acid --50% of theoretical yield [35], and H_2_ (dark fermentation) -- 50% of theoretical yield [38].

To better understand the limitations of microbial cell factories, we refer to an ancient Chinese philosophy: Yin-Yang. Yin-Yang describes both the bright side and dark side of an object in the world. Yin and Yang oppose each other but are also interdependent. In the case of metabolic engineering, the microbial metabolism is operated by thousands of enzymatic reactions and mass transport processes that involve both carbon (Yang) and energy (Yin) transformations (Figure 1). Through billions of years of evolution and environmental adaptations, biological systems have evolved closely interdependent carbon fluxes for biomass growth and energy fitness, which are similar to the intertwined Yin-Yang forces. Although it is easy to engineer microbial hosts to produce small amounts of diverse products, manufacturing a particular compound with titers and rates beyond the economic break-even point could be limited by suboptimal energy metabolism. In microbial conversions of a substrate to a product, metabolic entropy increases when carbon flux is redirected to the final products (Figure 3a & b). For example, Figure 3c shows the energy loss during conversion of glucose to different biofuels.

To leverage the “Yin-Yang” balance, metabolic engineers tried a few practical approaches to promote energy metabolism and boost productivity. For instance, *Vitreoscilla* hemoglobin (VHb), a soluble bacterial protein, has been used to enhance energy metabolism by improving oxygen delivery, which can significantly improve cell growth and increase chemical production under oxygen-limited conditions [39]. Furthermore, an energy-conserving pathway in *E. coli* was developed through metabolic evolution for high production of succinate from glucose fermentation [40]. This study indicates that the overexpression of a phosphoenolpyruvate carboxykinase increases the net production of ATP, compared to the primary mixed acid fermentation pathway via PEP carboxylase. The extra energy supply allows *E. coli* to produce succinate close to the theoretical maximum. In another case, an ATP-consuming reaction was introduced into *S. elongatus* PCC 7942 to drive carbon flux from acetyl-CoA to 1-butanol [41]. This study of 1-butanol production further validates that the ATP coupling reaction can make engineered pathways thermodynamically more favorable. To this end, we summarize the following suggestions to overcome the energy roadblocks.

First, a clear understanding of the entire carbon and energy metabolisms in microbial species would help us to conquer the energy limitations. Using *E. coli* as an example, ATP significantly impacts the product distributions at the pyruvate node [42]. Understanding ATP fluxes can offer rational design of *E. coli* strains for improving product biosynthesis [40,43]. Flux balance analysis (FBA) and ^13^C-metabolic flux analysis (MFA) are the only available tools that can quantify energy expenditures. FBA can characterize cell energy metabolism by dividing ATP cost into non-growth associated loss and growth-associated maintenance [22]. Due to the metabolic nature of suboptimal carbon fluxes, FBA, relying on the objective functions, may overestimate the cell potential for biosynthesis capability. ^13^C-MFA uses tracer experiments to constrain the FBA model so that it can precisely measure enzyme reaction rates. ^13^C-MFA can profile carbon fluxes through all energy generation/consumption pathways and deduce energy flows in the cell metabolism (ATP and cofactor balancing) [35]. Flux analysis not only allows us to determine the hidden Yin-Yang balance and to design rational engineering strategies, but also to characterize metabolic entropy and identify a strain’s energy potential for further improvement. Although ^13^C-MFA has not been widely accepted as a routine laboratory measurement tool to assess the engineered microbial hosts, this technology has excellent potential to reveal pathway engineering burdens (i.e., predict “the last straw” in genetic modifications). This tool can informatively tell metabolic engineers and project sponsors what can be done and what cannot be done.

Second, metabolic engineers need to exploit native pathways and avoid extensive pathway reconstruction. In history, many industrial successful cases of improved strain tolerance or productivity just relied on random mutation or evolution, leveraging Natural Selection of mutants for the best ‘Yin-Yang’. Additionally, efforts should aim product synthesis at pathways that do not require significant ATP expenditures (such as ethanol or organic acids). For example, the acetate overproduction pathway in *E. coli* generates abundant ATP, and the engineered strain performs very well even when its oxidative phosphorylation, TCA cycle and competing fermentation pathways are disrupted [43]. When microbial hosts have low-burden biosynthesis pathways, they show robustness in industrial processes. Moreover, artificial synthetic circuits, efflux pumps, or novel pathways should be carefully considered in terms of the energy penalty. By revealing the tradeoffs behind synthetic biology parts via flux analysis approach, metabolic engineers can rationally design their engineering strategies.

Thirdly, although it is difficult to break the Yin-Yang balance in a natural microorganism, synthetic biologists may re-program the carbon metabolism and energy “fitness” by engineering novel microbial systems. Metabolic engineers often apply pathway overexpression to improve the strain productivity. These practices typically encounter adverse metabolic shifts due to energy imbalances. However, the creation of a “minimal or smart” cell can remove unnecessary genes in microbial hosts in effort to reduce cell burden and unlock the biosynthesis regulations [44,45]. Additionally, synthetic biologists try to design and assemble cells using synthetic chromosomes [46]. These artificial biological systems might not necessarily follow the natural Yin-Yang balance evolved over billions of years, so they could have an unusually efficient energy metabolism, and thus achieve product yields close to the theoretical maximum.

Fourth, biological conversion can be integrated with non-living processes to reduce the biosynthesis burden. We can use robust microbial hosts to make simple molecules with high yields and titers, and then convert these molecules into a desired product with a complicated structure via biological and chemical processes. For example, the Keasling Lab achieved the total synthesis of artemisinin with a two-stage semi-synthetic approach. They used the mevalonate pathway in *Saccharomyces cerevisiae* to synthesize artemisinic acid, followed by a four-step chemical conversion of artemisinic acid to artemisinin [47]. The Zhang Lab has made biopolymers by using engineered *E. coli* as a first step, to produce a simple molecule mevalonic acid, and then chemically converting it into biopolymers [48]. A significant advantage of these integrated processes is an extremely efficient bioconversion in the first step using a short microbial pathway [49]. For instance, the titer of the semi-product mevalonic acid can reach as high as 88 g/L because its synthesis only requires three steps from the central metabolic node (acetyl-CoA) [48]. In another and more radical approach, an artificial cell-free system containing enzyme cocktails can mimic one or many functions of a biological system. Such systems can be used to synthesize products with near maximum theoretical yields [50,51] since they have no cell maintenance cost.

Lastly, development of non-model microbial workhorses with desired traits in energy metabolism (e.g., photosynthesis) may achieve higher biosynthesis potentials, enabling the design of industrial biorefineries for the production of a broad range of products. In fact, even in the modern era of genomics, it is estimated that > 99% of all bacterial species remain unknown [52]. Some non-model species might have a unique energetics that can facilitate product synthesis. For example, Algenol is developing the engineered cyanobacteria for phototrophic ethanol production from CO_2_ (http://www.algenol.com/). Moreover, cyanobacterial species have shown faster growth and higher production rate/titer by co-utilization of organic substrates [53]. Cyanobacterial photo-fermentations, using cheap feedstocks, CO_2_ and light energy, may facilitate cost-effective and large-scale biorefineries. In fact, Nature is the best synthetic biologist and may have already prepared us excellent chasses that we have not discovered yet. When we try to out-do Nature’s performance, we must first assimilate her lessons of ‘Yin-Yang’.

## Conclusions

We have discussed the Yin-Yang concept as the underlying regulatory mechanism in cell metabolism. Biosynthesis of diverse useful products requires sophisticated genetic pathway engineering to steer a high flux to the final product while energy fitness requires the cell metabolism to be wisely changed. Since the powerhouse in microbial cell factory is not limitless, energy shortage eventually leads to metabolic shifts and reduced cell productivity in engineered microbes. The Yin-Yang balance may caution against the assumption that the host metabolism can be modified extensively to produce any desired products. By using fluxomics, we can formulate guidelines to avoid many false starts and dead ends during metabolic engineering. In addition, industrial bioprocess always faces numerous constraints and trade-offs (mass transfer limitations in fermentation, sterilization, strain stability, contaminations, and aeration costs). Feedstock selections, downstream product separation, and waste treatment are critical issues that impact product profitability. Thus, the design-build-test-learn cycle should cover both strain development and economic analysis. Nevertheless, the Yin-Yang philosophy provides general insights into all biotechnology tradeoffs.

## References

[1] Sun N, Zhao H (2013). Transcription activator-like effector nucleases (TALENs): a highly efficient and versatile tool for genome editing. Biotechnol Bioeng.

[2] Jiang W, Bikard D, Cox D, Zhang F, Marraffini LA (2013). RNA-guided editing of bacterial genomes using CRISPR-Cas systems. Nat Biotech.

[3] Pratt AJ, MacRae IJ (2009). The RNA-induced silencing complex: a versatile gene-silencing machine. J Biol Chem.

[4] Qi LS, Larson MH, Gilbert LA, Doudna JA, Weissman JS, Arkin AP (2013). Repurposing CRISPR as an RNA-guided platform for sequence-specific control of gene expression. Cell.

[5] Wang HH, Isaacs FJ, Carr PA, Sun ZZ, Xu G, Forest CR (2009). Programming cells by multiplex genome engineering and accelerated evolution. Nature.

[6] Isaacs FJ, Carr PA, Wang HH, Lajoie MJ, Sterling B, Kraal L (2011). Precise manipulation of chromosomes *in Vivo* enables genome-wide codon replacement. Science.

[7] Atsumi S, Higashide W, Liao JC (2009). Direct photosynthetic recycling of carbon dioxide to isobutyraldehyde. Nat Biotech.

[8] Lindberg P, Park S, Melis A (2010). Engineering a platform for photosynthetic isoprene production in Cyanobacteria, using *Synechocystis* as the model organism. Metab Eng.

[9] Oliver JW, Machado IM, Yoneda H, Atsumi S (2013). Cyanobacterial conversion of carbon dioxide to 2,3-butanediol. Proc Natl Acad Sci U S A.

[10] Nielsen J, Fussenegger M, Keasling J, Lee SY, Liao JC, Prather K (2014). Engineering synergy in biotechnology. Nat Chem Biol.

[11] Hoehler TM, Jorgensen BB (2013). Microbial life under extreme energy limitation. Nat Rev Micro.

[12] Pfeiffer T, Schuster S, Bonhoeffer S (2001). Cooperation and competition in the evolution of ATP-producing pathways. Science.

[13] MacLean RC, Gudelj I (2006). Resource competition and social conflict in experimental populations of yeast. Nature.

[14] Ibarra RU, Edwards JS, Palsson BO (2002). *Escherichia coli* K-12 undergoes adaptive evolution to achieve *in silico* predicted optimal growth. Nature.

[15] Fischer E, Sauer U (2005). Large-scale *in vivo* flux analysis shows rigidity and suboptimal performance of *Bacillus subtilis* metabolism. Nat Genet.

[16] Birnbaum S, Bailey JE (1991). Plasmid presence changes the relative levels of many host cell proteins and ribosome components in recombinant *Escherichia coli*. Biotechnol Bioeng.

[17] Wang Z, Xiang L, Shao J, Wegrzyn A, Wegrzyn G (2006). Effects of the presence of ColE1 plasmid DNA in *Escherichia coli* on the host cell metabolism. Microb Cell Fact.

[18] Ow DS-W, Lee D-Y, Yap MG-S, Oh SK-W (2009). Identification of cellular objective for elucidating the physiological state of plasmid-bearing *Escherichia coli* using genome-scale *in silico* analysis. Biotechnol Prog.

[19] Stephanopoulos G, Aristidou A, Nielsen J (1998). Metabolic Engineering: Principles and Methodologies.

[20] Taniguchi Y, Choi PJ, Li G-W, Chen H, Babu M, Hearn J (2010). Quantifying E. coli Proteome and transcriptome with single-molecule sensitivity in single cells. Science.

[21] Heyland J, Blank LM, Schmid A (2011). Quantification of metabolic limitations during recombinant protein production in *Escherichia coli*. J Biotechnol.

[22] Varma A, Palsson BO (1994). Stoichiometric flux balance models quantitatively predict growth and metabolic by-product secretion in wild-type *Escherichia coli* W3110. Appl Environ Microbiol.

[23] Sauer U, Bailey JE (1999). Estimation of P-to-O ratio in *Bacillus subtilis* and its influence on maximum riboflavin yield. Biotechnol Bioeng.

[24] Xiao Y, Feng X, Varman AM, He L, Yu H, Tang YJ (2012). Kinetic Modeling and isotopic investigation of isobutanol fermentation by two engineered *Escherichia coli* strains. Ind Eng Chem Res.

[25] Atsumi S, Wu TY, Machado IMP, Huang WC, Chen PY, Pellegrini M (2010). Evolution, genomic analysis, and reconstruction of isobutanol tolerance in *Escherichia coli*. Mol Syst Biol.

[26] Dunlop MJ, Dossani ZY, Szmidt HL, Chu HC, Lee TS, Keasling JD (2011). Engineering microbial biofuel tolerance and export using efflux pumps. Mol Syst Biol.

[27] Foo JL, Jensen HM, Dahl RH, George K, Keasling JD, Lee TS (2014). Improving Microbial Biogasoline Production in Escherichia coli Using Tolerance Engineering. mBio.

[28] Zhang Y-HP, Lynd LR (2005). Cellulose utilization by *Clostridium thermocellum*: bioenergetics and hydrolysis product assimilation. Proc Natl Acad Sci USA.

[29] Alper H, Moxley J, Nevoigt E, Fink GR, Stephanopoulos G (2006). Engineering yeast transcription machinery for improved ethanol tolerance and production. Science.

[30] Lam FH, Ghaderi A, Fink GR, Stephanopoulos G (2014). Engineering alcohol tolerance in yeast. Science.

[31] Xu M, Zhao J, Yu L, Tang IC, Xue C, Yang S-T (2015). Engineering *Clostridium acetobutylicum* with a histidine kinase knockout for enhanced n-butanol tolerance and production. Appl Microbiol Biot.

[32] Orth J, Conrad T, Na J, Lerman J, Nam H, Feist A (2011). A comprehensive genome-scale reconstruction of *Escherichia coli* metabolism-2011. Mol Syst Biol.

[33] Lennen RM, Kruziki MA, Kumar K, Zinkel RA, Burnum KE, Lipton MS (2011). Membrane stresses induced by overproduction of free fatty acids in *Escherichia coli*. Appl Environ Microb.

[34] Steen EJ, Kang Y, Bokinsky G, Hu Z, Schirmer A, McClure A (2010). Microbial production of fatty-acid-derived fuels and chemicals from plant biomass. Nature.

[35] He L, Xiao Y, Gebreselassie N, Zhang F, Antoniewicz MR, Tang YJ (2014). Central metabolic responses to the overproduction of fatty acids in *Escherichia coli* based on ^13^C-metabolic flux analysis. Biotechnol Bioeng.

[36] Orth J, Palsson B (2012). Gap-filling analysis of the iJO1366 *Escherichia coli* metabolic network reconstruction for discovery of metabolic functions. BMC Syst Biol.

[37] Atsumi S, Hanai T, Liao JC (2008). Non-fermentative pathways for synthesis of branched-chain higher alcohols as biofuels. Nature.

[38] Rachman MA, Furutani Y, Nakashimada Y, Kakizono T, Nishio N (1997). Enhanced hydrogen production in altered mixed acid fermentation of glucose by *Enterobacter aerogenes*. J Ferment Bioeng.

[39] Wei X-X, Chen G-Q. Chapter Fifteen - Applications of the VHb Gene vgb for Improved Microbial Fermentation Processes. In Method Enzymol. Volume Volume 436. Edited by Robert KP: Academic Press; 2008: 273–287 10.1016/S0076-6879(08)36015-7 18237638

[40] Zhang X, Jantama K, Moore JC, Jarboe LR, Shanmugam KT, Ingram LO (2009). Metabolic evolution of energy-conserving pathways for succinate production in E*scherichia coli*. Proc Natl Acad Sci.

[41] Lan EI, Liao JC (2012). ATP drives direct photosynthetic production of 1-butanol in cyanobacteria. Proc Natl Acad Sci.

[42] Wang Q, Ou MS, Kim Y, Ingram LO, Shanmugam KT (2010). Metabolic flux control at the pyruvate node in an anaerobic *Escherichia coli* strain with an Active Pyruvate Dehydrogenase. Appl Environ Microb.

[43] Causey TB, Zhou S, Shanmugam KT, Ingram LO (2003). Engineering the metabolism of *Escherichia coli* W3110 for the conversion of sugar to redox-neutral and oxidized products: Homoacetate production. Proc Natl Acad Sci.

[44] Forster AC, Church GM (2006). Towards synthesis of a minimal cell. Mol Syst Biol.

[45] Trinh CT, Unrean P, Srienc F (2008). Minimal Escherichia coli cell for the most efficient production of ethanol from Hexoses and Pentoses. Appl Environ Microb.

[46] Gibson DG, Glass JI, Lartigue C, Noskov VN, Chuang R-Y, Algire MA (2010). Creation of a bacterial cell controlled by a chemically synthesized genome. Science.

[47] Paddon CJ, Keasling JD (2014). Semi-synthetic artemisinin: a model for the use of synthetic biology in pharmaceutical development. Nat Rev Micro.

[48] Xiong M, Schneiderman DK, Bates FS, Hillmyer MA, Zhang K (2014). Scalable production of mechanically tunable block polymers from sugar. Proc Natl Acad Sci.

[49] Colletti PF, Goyal Y, Varman AM, Feng X, Wu B, Tang YJ (2011). Evaluating factors that influence microbial synthesis yields by linear regression with numerical and ordinal variables. Biotechnol Bioeng.

[50] Hodgman CE, Jewett MC (2012). Cell-free synthetic biology: thinking outside the cell. Metab Eng.

[51] Ye X, Wang Y, Hopkins RC, Adams MWW, Evans BR, Mielenz JR (2009). Spontaneous high-yield production of hydrogen from cellulosic materials and water catalyzed by enzyme cocktails. ChemSusChem.

[52] Lasken RS, McLean JS (2014). Recent advances in genomic DNA sequencing of microbial species from single cells. Nat Rev Genet.

[53] You L, Berla B, He L, Pakrasi HB, Tang YJ (2014). ^13^C-MFA delineates the photomixotrophic metabolism of *Synechocystis sp.* PCC 6803 under light- and carbon-sufficient conditions. Biotechnol J.

